# Supplementary material for: 300-Gbps optical interconnection using neural-network based silicon microring modulator
Source: Commun Eng. 2023 Sep 23;2:67. doi: 10.1038/s44172-023-00115-x (PMC10955910; doi:10.1038/s44172-023-00115-x)
Supplement: Supplementary file 2 — Supplemental Information [file 44172_2023_115_MOESM2_ESM.pdf]

Supplementary Information

300-Gbps Optical Interconnection using Neural-Network based Silicon Microring Modulator

Fangchen Hu,[1,5] Yuguang Zhang,[2,5] Hongguang Zhang,[2,5] Zhongya Li,[1] Sizhe Xing,[1] Jianyang Shi,[1] Junwen Zhang,[1*] Xi Xiao,[3,4,*] Nan Chi,[1,*] Zhixue He,[3,4] and Shaohua Yu[3,4]

[1] Key Laboratory for Information Science of Electromagnetic Waves (MoE), Fudan University, Shanghai 200433, China.

[2]National Information Optoelectronics Innovation Center, Wuhan 430074, China.

[3]State Key Laboratory of Optical Communication Technologies and Networks, China Information Communication Technologies Group Corporation, Wuhan 430074, China.

[4]Peng Cheng Laboratory, Shenzhen 518055, China.

[5]These authors contributed equally: Fangchen Hu, Yuguang Zhang, Hongguang Zhang.

*Corresponding author. Email: junwenzhang@fudan.edu.cn, nanchi@fudan.edu.cn, xxiao@wri.com.cn.

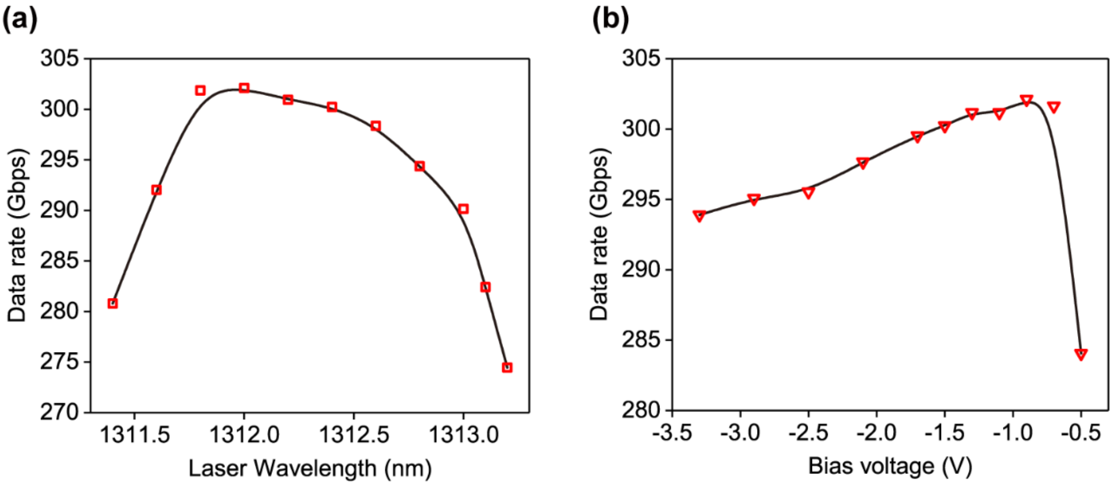

Supplementary Fig. S1 Optimization in working parameters of depletion-mode Si-MRM for high-speed optical interconnection. (a) Data rate versus different laser wavelengths. (Vpp of signal is 0.6 V, bias voltage is -0.9 V) (b). Data rate versus different bias voltages. (Vpp of signal is 600 mV, laser wavelength is 1312 nm. The optimal bias voltage and laser wavelength is -0.9 V and 1312 nm, respectively.

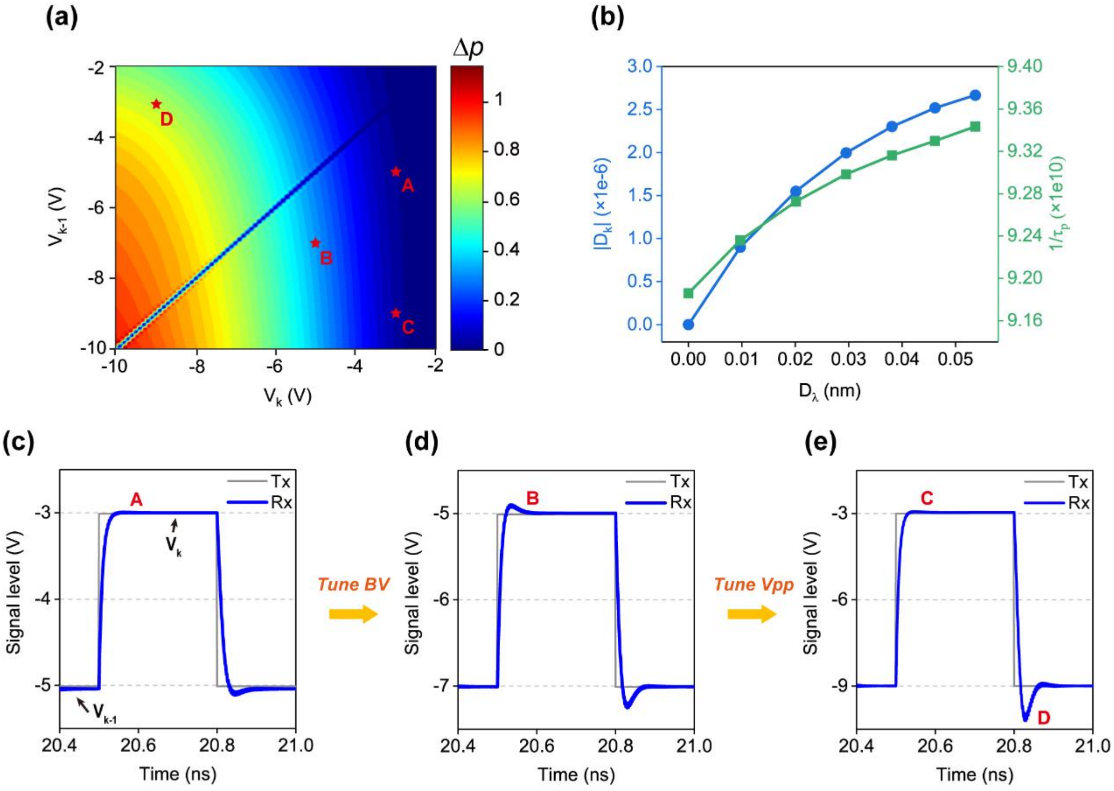

Supplementary Fig. S2 Simulation results of the time-relevant dependency of transient nonlinearity on wavelength detuning. (a) The normalized amplitude of overshoot (Δp) versus levels of input electrical signal in the *(k-1)*$_{th}$ (V_{k-1}) and k_{th} (V_k) time slots, respectively. (b) The absolute value of transient response term ($|D_k|$) and reciprocal of cavity photon lifetime ($\frac{1}{\tau_p} = \frac{2}{\tau}$) versus different wavelength detuning (D_λ). (c-e) The waveforms of output optical signal when Si-MRM is modulated three set of NRZ signal with different bias voltages and Vpps. Their signal level distribution matches

the point A-D markered in (a). The adjustment of bias voltage and Vpp can change D_{λ}, which brings the change in Δp. Their differences of Δp are also observed from the signal waveform intuitively.

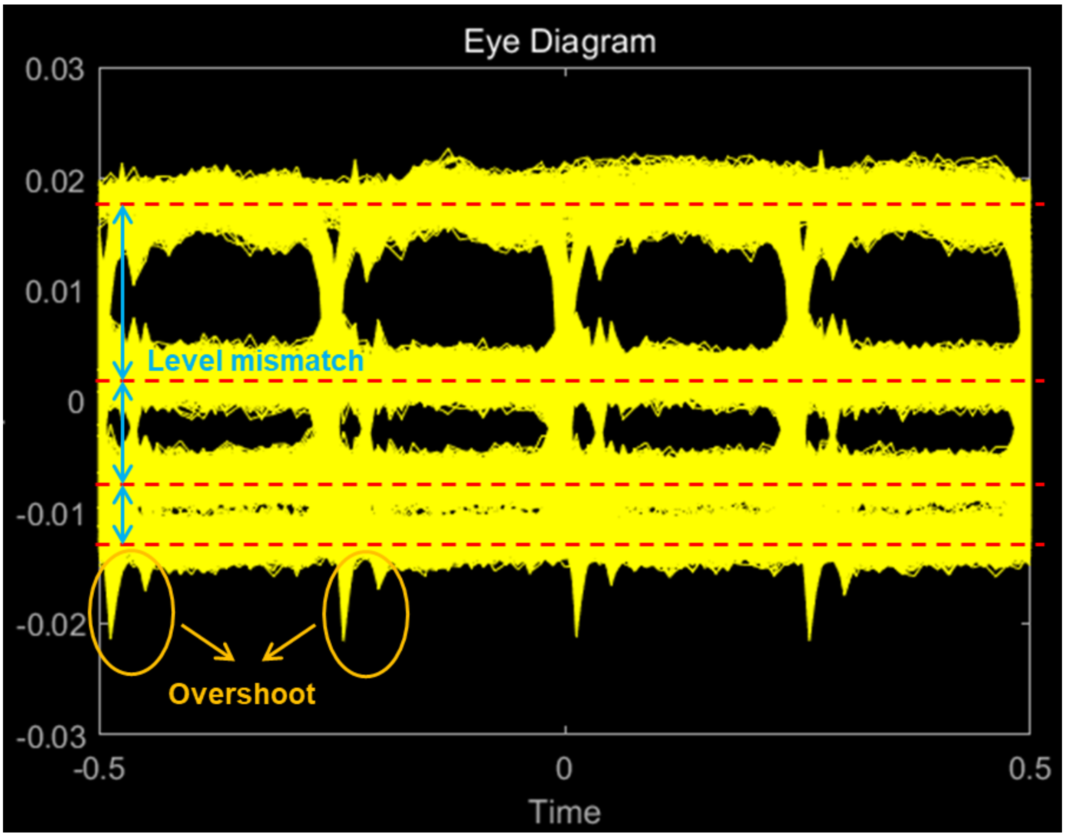

Supplementary Fig. S3 The eye diagram of received 5GBaud PAM4 electrical signal obtained from the experiment. The static and transient nonlinear signal distortion are observed. The level mismatch comes from static modulation nonlinearity of Si-MRMs. The overshoot at the level with high wavelength detuning originate from transient modulation nonlinearity of Si-MRMs.

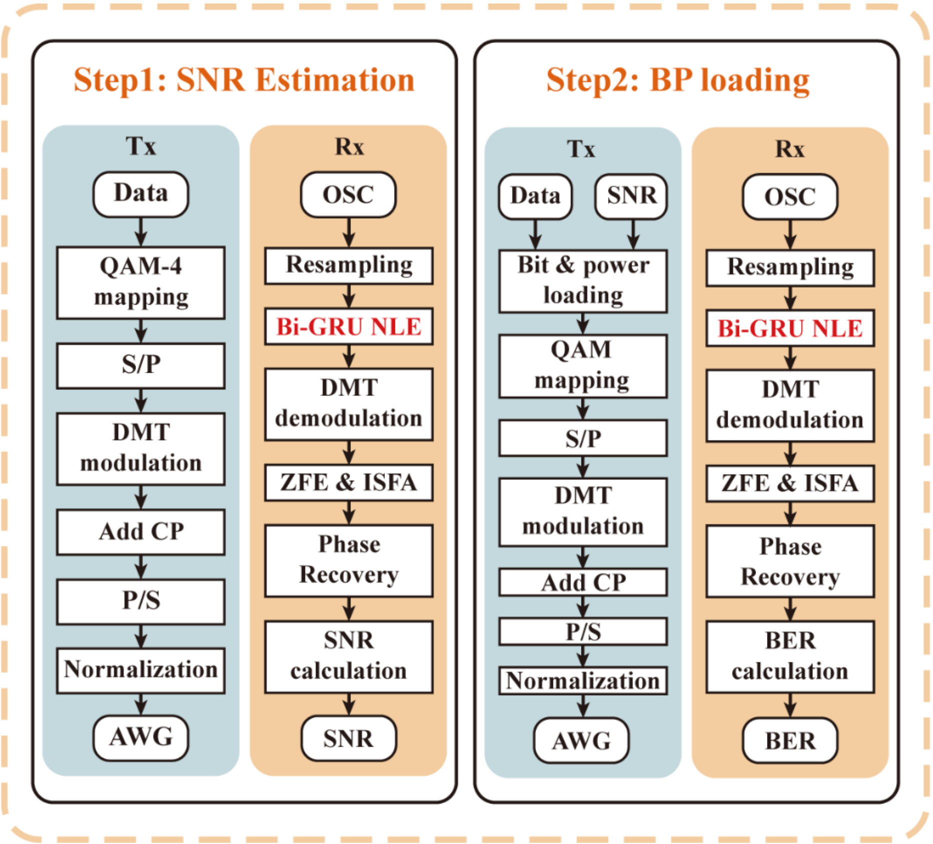

Supplementary Fig. S4 Flow chart and principle of the DSP algorithm in the experiment. The basic signal modulation format is discrete multi-tone modulation. To maximize the transmission speed, the bit and power (BP) loading technology is used to maximize the loaded information source entropy. Before the BP loading, SNR estimation is essential to obtain the channel information for generation of accurate BP loading strategy. The modified Bi-GRU serves as a nonlinear equalizer (NLE) applied after the resampling operation on the received signal.

Supplementary Note 1: Analysis on the transient modulation nonlinearity and its dependency.

The key step in simulating the Si-MRM is to obtain the correlation of n_{eff}, τ_e and τ_l with the input voltage. They can be extracted by using the high-order polynomial expansion to fit the transmission spectra of the Si-MRM at different input voltages. The transmission spectrum $T(V)=\left|\frac{j(\omega-\omega_{res}(V))+\frac{1}{\tau_l(V)}-\frac{1}{\tau_e(V)}}{j(\omega-\omega_{res}(V))+\frac{1}{\tau_l(V)}+\frac{1}{\tau_e(V)}}\right|^2$ [1]. The transmission spectrum is determined by the structure and doping concentration of the P-N junction in the Si-MRM.[35] In our simulation, the ring radius, doping concentration of N, doping concentration of P and junction feature length are 10 μm, 8.2e^{24} (1/cm^3), 3.5e^{24} (1/cm^3) and 200 nm, respectively. By fitting the transmission spectrum, the correlations of n_{eff}, τ_e and τ_l with the input voltages are:

$$n_{eff}=-1.3686e^{-10}V^4-3.4678e^{-9}V^3-3.6345e^{-8}V^2-3.1326e^{-7}V+0.0208 \quad \text{(S1)}$$

$$\tau_e=-2.661e^{-15}V^3-5.7557e^{-14}V^2-1.3669e^{-12}V+6.2055e^{-11} \quad \text{(S2)}$$

$$\tau_l=4.1324e^{-15}V^3+9.1925e^{-14}V^2+9.2863e^{-12}V+3.4369e^{-11} \quad \text{(S3)}$$

In the Supplementary Fig. S1(a), we investigate the dependence of Δp on the signal levels V_{k-1} and V_k at (k-1)th and kth time slot with a tight time increment (~20 ps), respectively. The amplitude ranges of Δp is normalized to [0, 1]. The Δp in the diagonal is zero because $|D_k|$ in the equation (s) of main text is zero when the signal levels of two adjacent time slots are constant. In the other region of the contour map, the value of Δp increases with increasing absolute values of V_k and V_{k-1}. The signal level pair (V_{k-1}, V_k) at the bottom-left corner of the contour map has the highest Δp

due to a relatively larger frequency detuning than other level pairs. The increase in frequency detuning ($D_\lambda = |\omega - \omega_{res}|$) leads to the variation in the transient nonlinear response $|D_k|$ which eventually intensifies Δp on the optical signal. If V_{k-1} is fixed at -2 V and V_k decreases from -2 V to -8 V, the gradually increased D_λ raises $|D_k|$ from 0 to 2.67e^{-6}, as shown in Supplementary Fig. S1(b), resulting in a larger Δp. This result explicitly demonstrates the positive impact of $|D_k|$ on Δp. Overall, the shift of electrical signals on a Si-MRM will lead to variation in the amplitude and phase of the stored optical energy in the ring resonator by changing frequency detuning. However, this variation is not abrupt, and reflects as a period of oscillation before a steady resonance state is again reached. Larger frequency (wavelength) detuning for a larger negative signal level pair will generate more serious oscillation manifesting a larger overshoot on the signal edge.

The bias voltage and Vpp of the electrical signal need to be finely adjusted to balance the transient nonlinear impairments, modulation depth and RoP to obtain a high modulation data rate. The bias voltage is usually set to the point where the resonance wavelength is far away the laser wavelength to get sufficient RoP, but accordingly will intensify the overshoot, as illustrated in Supplementary Fig. S1(c-d). When the bias voltage decreases from -4 V to -6 V, point B, representing the signal level pair (-7 V, -5 V), has a higher Δpd than point A, representing the level pair (-5 V, -3 V). If the bias voltage is fixed, increasing the Vpp can increase the modulation depth, but amplifies the overshoot at the signal level at which V_k is decreased (point D) but relieves the overshoot at the signal level at which V_k is increased (point C) as shown in

Supplementary Fig. S1 (e). It's noted that V_k has a higher impact than V_{k-1} on the variation in Δp. This is why point D has the largest Δp because V_k currently has the lowest value (-9 V) though V_{k-1} is high (-3 V). The static nonlinear distortion is not present in Supplementary Fig. S1 (c-e) because all levels of input and output signals are scaled to a similar level for intuitive comparison of overshoots.

Supplementary Note 2: The inner structure of a typical GRU cell

The inner structure of a typical GRU cell consists of nonlinear activation functions and three kinds of gates: update gate, reset gate and output gate. Taking the forward GRU cell at the *k*-th as an example, the update gate controls how much the previous memory states (C_{k-1}^{f}) and current input (O_k) could join the update of the current memory state (C_k^f) by the parameter W_z. The reset gate determines whether the current memory state ignores the previous memory states and even resets with the current input only, which depends on the parameter W_r. The output gate finally calculates the current memory state that goes back as the input of the GRU cell in the next time slot. The outputs of the three gates are given as follows [2]:

$$\begin{aligned}
&\textit{Update gate}: \overrightarrow{a_k} = s\left(W_z\left[\overrightarrow{O_k}, \overrightarrow{C_{k-1}^f}\right]\right) \\
&\textit{Reset gate}: \overrightarrow{b_k} = s\left(W_r\left[\overrightarrow{O_k}, \overrightarrow{C_{k-1}^f}\right]\right) \\
&\textit{Output gate}: \overrightarrow{C_k^f} = \left(1-\overrightarrow{a_k}\right)\odot\overrightarrow{C_{k-1}^f} + \overrightarrow{a_k}\odot\overrightarrow{T_k^f} \\
&\overrightarrow{T_{k-1}^f} = \kappa\left(W_a\left[\overrightarrow{O_k}, \overrightarrow{b_k}\odot\overrightarrow{C_{k-1}^f}\right]\right)
\end{aligned}$$

where W_z, W_r and W_a are the parameters to be updated in the training process. s and κ represent the sigmoid and tanh activation functions, respectively. The operators [] and $\odot$ represent cascade and multiplication between elements in the vector, respectively.

Supplementary References:

1 Shin, M. *et al.* A Linear Equivalent Circuit Model for Depletion-Type Silicon Microring Modulators. *IEEE Trans Electron Devices.* **64**, 1140-1145 (2017).

2 Cho, K. *et al.* Learning phrase representations using RNN encoder-decoder for statistical machine translation. *arXiv preprint arXiv:1406.1078* (2014).
